# Chemical profiling, biostatic and biocidal dynamics of *Origanum vulgare* L. essential oil

**DOI:** 10.1186/s13568-019-0764-y

**Published:** 2019-03-26

**Authors:** Sahar Fikry, Noha Khalil, Osama Salama

**Affiliations:** 1 Faculty of Allied Medical Sciences, Pharos University in Alexandria, Alexandria, 21311 Egypt; 2 Faculty of Pharmaceutical Sciences and Pharmaceutical Industries, Future University in Egypt, Cairo, 11835 Egypt

**Keywords:** *Origanum vulgare*, Essential oil, GC/MS, Terpinen-4-ol, Biostatic, Biocidal dynamics

## Abstract

*Origanum vulgare* L. (Lamiaceae) is a widespread flavoring culinary and medicinal herb. The present study aimed at investigating the antimicrobial activity of *Origanum vulgare* (OV) essential oil (EO) through illustrating its biostatic, biocidal and the dynamics of the biocidal activity against 11 different microorganisms. GC/MS of OV EO allowed the identification of 32 compounds representing 99.94% of the oil. The two major identified compounds were terpinen-4-ol (38.35%) and *trans*-sabinene hydrate (10.06%). Different methods were employed to illustrate the biostatic activity of OV EO. Results of the biostatic studies on OV EO using agar and broth dilution methods showed that *Staphylococcus aureus* (*S. aureus*) was the most sensitive organism; with a Minimum inhibitor concentration (MIC) 1.18 mg/ml. Agar diffusion method showed that the highest activity was observed against *Bordetella bronchiseptica* (*Br. bronchiseptica*), *Saccharomyces cerevisiae* (*S. cerevisiae*), *Bacillus subtilis* (*B. subtilus*) and *Staphylococcus epidermidis* (*S. epidermidis*) with inhibition zones 38 ± 1.5, 29.5 ± 0.8, 26.9 ± 0.9 and 26.9 ± 1.1 mm, respectively. Studying the dynamics of 1% v/v OV essential oil emulsion over a period of 6 h revealed that *Escherichia coli* (*E. coli*)*, B. subtilis, S. epidermidis* and *S. cerevisiae* had the fastest response. Also increasing concentrations of OV oil emulsion increased the rate of cell killing and the duration of growth lag phase increased correspondingly. These data indicated that OV EO produces a concentration and time-dependent antimicrobial activity.

## Introduction

Some medicinal and aromatic plants (MAPs) are a rich source of essential oils, which have proven to possess a wide variety of biological activities such as antimicrobial, anti-inflammatory, antiseptic, anticancer, analgesic and sedative effects (Bhalla et al. 2013; Dhifi et al. 2016). *Origanum vulgare* L. (OV), which is also known as oregano or marjoram is a widespread flavoring culinary herb belonging to family Lamiaceae (Kokkini et al. 2003). Traditionally, the plant has been employed as a remedy for different ailments like gastrointestinal disorders, colds, whooping and convulsive coughs, menstrual problems, headaches, depression and pruritis (Fleisher and Sneer 1982; Krishnakumar and Potty 2012). The plant’s essential oil and different extracts have been reported as antioxidant, antimicrobial, anti-inflammatory as well as hypolipidemic (Bhat et al. 2018; Elshafie et al. 2017; Leyva-López et al. 2017; Milos et al. 2000; Morshedloo et al. 2018; Soliman et al. 2016). The essential oil composition varied widely according to the geographical area and growth conditions (Gong et al. 2014; Hernández-Hernández et al. 2014; Morshedloo et al. 2017). Studies show that OV EO mostly contains phenolic compounds, mono-and sesquiterpene hydrocarbons, as well as their oxygenated derivatives. Major identified compounds in Chinese OV EO were thymol (42.9%) and *p*-cymen-2-ol (37.5%), while OV EO collected in Pakistan contained mainly β-citronellol (78.7%) and citronellol acetate (5.9%) (Gong et al. 2014). Essential oil of Saudi Arabia OV contained mainly carvacrol (70.2%) and γ-terpinene (5.6%) (Khan et al. 2018). On the other hand, Mexican OV EO had major its major compounds thymol (66.3%) and γ-terpinene (9.6%) (Hernández-Hernández et al. 2014) and the Brazilian one contained thymol (21.9%) and carvacrol (4.7%) as major constituents (Pradebon Brondani et al. 2018). OV cultivated in Spain contained in its essential oil *cis*-sabinene hydrate (37%) and terpinene-4-acetate (16.2%). This compositional variation may be attributed to difference in growth conditions, origin of plant collection, stage of plant maturity, physiological modifications in response to various environmental factors and stresses, harvesting time, drying methods, method of essential oil isolation or even the solvents used for the GC/MS analysis (Arranz et al. 2015; Moghaddam and Mehdizadeh 2017).

Recently, there has been a growing interest in the use of crude essential oils or their pure isolated compounds in the food and pharmaceutical industries, either as preservatives or antimicrobial agents (Herman et al. 2013; Prakash et al. 2012). They are being relied on as natural alternatives rather than the use of synthetic chemical agents; which cause multidrug resistance and several side effects. This is due to their constituents of bioactive compounds which possess both antioxidant and antimicrobial properties. Essential oils of different *Origanum* species have demonstrated good antimicrobial effects against various bacteria including *S. aureus, Salmonella* spp., *Pseudomonas* and *E. coli*, and fungi including *Candida albicans* and *Aspergillus* spp. (Bhat et al. 2018; Carneiro de Barros et al. 2009; Chaves-Lopez et al. 2012; Coccimiglio et al. 2016; de Barros et al. 2012; Kacaniova et al. 2017). The oil is known to be rich in compounds with strong antimicrobial activity like thymol, carvacrol, γ-terpinene, as well as *cis*- and *trans*-sabinene hydrate (Didry et al. 1993; Lee et al. 2013; Magi et al. 2015; Sharifi-Rad et al. 2018). Standardized reliable methods are essential for the elucidation of the antimicrobial activity and therapeutic potential of essential oils.

The present study aimed at investigating the in vitro antimicrobial activity of OV EO through illustrating its biostatic, biocidal and the dynamics of the biocidal activity against 11 different American Type Culture Collection (ATCC) microbial strains microorganisms. This was in addition to chemical profiling of the essential oil using GC/MS technique in order to relate the antimicrobial activity to the essential oil composition.

## Materials and methods

### Plant material

*Origanum vulgare* (OV) plants were collected from the medicinal farm of Arab Company for Pharmaceuticals and Medicinal plants (Mepaco. Medifood, Egypt). Plants identity was authenticated by Prof. Mohamed El-Gebaly, professor of plant taxonomy, at the Department of Botany of the National Research Center in Egypt.

### Extraction of OV EO

Dried aerial parts of *O. vulgare* were subjected to hydro-distillation using a Clevenger-type apparatus, for 4 h. The obtained essential oil was dried over anhydrous sodium sulfate. The oil was kept refrigerated in a sealed amber vial till use.

### Identification of OV EO compounds using GC/MS

An Agilent 7890A gas chromatograph (Agilent Technologies, Palo Alto, CA, USA) equipped with a RTX-5MS capillary column (30 m × 0.32 mm, film thickness 0.25 μm) was used for the GC–MS analysis of OV EO. The apparatus was coupled to an Agilent 5975C (Agilent Technologies, Palo Alto, CA, USA) mass selective detector. Initial oven temperature was kept at 40 °C for 2 min. Temperature was then raised at a rate of 5 °C/min till reaching 210 °C. Temperatures of both the injector and detector were adjusted at 290 and 300 °C, respectively. Helium was used as a carrier gas with a flow rate of 2 ml/min. OV oil sample (0.1 μl) was injected manually in the split mode. Mass spectra were recorded in EI mode and 35–500 m/z range with ionization voltage, 70 eV. Ion source temperature was set at 230 °C. Kovat’s index was calculated for all compounds using a homologous series of *n*-alkanes (C_5_–C_24_) using the same operating conditions. Identification of different oil compounds was based on comparison of the obtained spectra with those available from MS libraries (Wiley) and by comparison of their experimentally determined Kovat’s index (KI) with those reported in the literature (Adams 2007). Peak areas were used for quantization of relative percentages of identified compounds.

### Antimicrobial studies

#### Preparation of sterile OV EO

OV EO emulsion (10% v/v) was prepared by thoroughly triturating ten volumes of the oil with one volume of Tween 20 (10 ml oil + 1 ml Tween 20). The total volume was completed to 100 with distilled water. The resultant emulsion was sterilized by filtration though a 0.45 µm membrane filter (Millipore, USA) and refrigerated in a sealed amber vial till use.

#### Source of microorganisms

The antimicrobial activity of OV EO was evaluated using laboratory reference strains ATCC for bacteria and fungi; which were purchased from IMTECH, Chandigarh, India. The tested microorganisms are listed in Table 3.

#### Culture media

Tryptic Soybean-Casein Digest Broth (TSB), Tryptic Soybean-Casein Digest Agar (TSA), Sabouraud Dextrose Broth (SDB) and Sabouraud were used as culture media and were sterilized according to the directions of the manufacturer (Sigma, USA).

#### Preparation of microbial inocula

- Vegetative bacterial strains: were grown in TSB at 37 °C for 18 h and diluted 1:100 with sterile TSB before use.
- Yeast strains: were grown in SDB ate 25 °C for 48 h and used undiluted.
- Bacterial spores: *B. subtilis* culture was heavily streaked onto TSA plate and incubated ate 37 °C for 5 days. The bacterial growth was scrapped off using sterile saline, vortexed and centrifuged for 5 min at 300 rpm. The pellet was suspended in sterile saline, vortexed and re-centrifuged. The resultant washed pellet was re-suspended in sterile saline and heated for 30 min in a water-bath at 80 °C to kill any vegetative cells. The resultant spore suspension was used as inoculum to study the dynamics of the biocidal activity of OV EO.
- Fungal spores: *A. niger* culture was grown as heavy streaks on SDA plate and incubated at 25 °C for 5 days. The developed spores were recovered using sterile saline containing 0.005% v/v Tween 20. The resultant spore suspension was vortexed and centrifuged for 5 min at 3000 rpm. The spore deposit was washed by dispersion in sterile saline-Tween, vortexed and re-centrifuged. The recovered washed spores were suspended in sterile saline-Tween and used as inoculum to study the biostatic activity of OV essential oil.

### Biostatic activity of OV EO

#### Agar dilution method

Ten ml of stock OV EO emulsion (10% v/v) were twofold serially diluted with distilled water. Each of the oil dilution was thoroughly mixed with an equal volume of double-strength sterile molten TSA or SDA maintained at 50 °C in a water bath. Portions of the TSA-oil or SDA-oil were poured onto sterile petri dishes, allowed to solidify and left to dry at room temperature for 30 min. Ten microliter (ca 10^5^ CFU/ml) aliquots of each of the prepared microbial inocula were transferred into the surface of the TSA-oil or SDA-oil plates as indicated. Inoculated TSA plates were than incubated for 24 h at 37 °C while the SDA plates were kept for 2–5 days at 25 °C. MIC of the oil was determined for each organism by visual inspection of the plates. MIC was considered as the lowest oil concentration giving no visible microbial growth; 1–2 colonies were neglected. Average of 3 results was recorded (Griffin et al. 2000).

#### Broth dilution method

Broth dilution method was performed according to Clinical and Laboratory Standards Institute (CLSI 2012). Ten ml of stock OV EO emulsion (10% v/v) were twofold serially diluted with sterile distilled water. Each oil dilution was mixed with equal volume of double strength sterile TSB or SDB and inoculated with 100 µl/5 ml mixture of the test inoculum followed by thorough mixing. Cultures were adjusted to 0.5 McFarland standard which contains approximately 1 to 2 × 10^8^ CFU/ml with tested bacterial strains, then dilute the 0.5 McFarland suspension 1:10 in sterile broth or saline to obtain a concentration of 10^7^ CFU/ml, the adjusted suspensions for final inoculation should be used within 15 min of preparation. One McFarland standard (equivalent to 1.5 × 10^8^ CFU/ml) is used for fungal strains. These systems were then incubated at 37 °C for 24 h and 25 °C for 48 h for bacteria and yeasts, respectively. Controls lacking the oil were also included. Visual inspection of the developed turbidity of the microbial growth was carried out and MIC of the oil was determined. Turbidity due to the oil emulsion was obvious only at ≥ 5.0% v/v. Average of 3 results was recorded.

#### Agar-well diffusion method

Fifty ml portions of molten sterile TSA or SDA maintained at 50 °C were inoculated, each with 100 µl of properly diluted inoculum and mixed well. Inoculated medium was poured into sterile petri dish (ca.15 cm id.) and allowed to solidify. Wells, each of 6 mm diameters were removed leaving empty wells. These were 3/4 filled with the OV EO or 1% v/v OV EO emulsion. The plates were allowed to stand at room temperature for 2 h and then incubated at 37 °C for 18 h and at 25 °C for 48 h in case of bacteria and yeast, respectively. The resultant inhibition zones were measured and the average values deduced (Balouiri et al. 2016). Average of 10 readings was recorded. Ampicillin, Ciprofloxacin and Amphotericin B were used as standard antimicrobial agents.

#### Radiant giant colony method

In case of *A. niger*, the radiant giant colony technique was used instead of the viable count technique because fungal spores were partially sensitive to the oil. SDA-oil plates containing 0–10% v/v of OV oil emulsion were prepared as described under “Agar dilution method”. Ten microliter volume of the prepared *A. niger* spore suspension were transferred onto the centers of the oil plates, allowed to stand at room temperature for 30 min and then incubated at 25 °C for 5 days. The diameters of the developed radiating giant colonies of the fungus were accurately measured (Daferera et al. 2000).

#### Calculation of colony count reduction

The count of each colony/plate containing specified oil concentration was divided by that of the control lacking the oil and multiplied by 100 to obtain percent colony count reduction vs. oil concentration. The obtained values were plotted against the corresponding log OV EO concentration (Fekrazad et al. 2014).

### Biocidal activity of OV EO

#### Viable count technique

Each reaction mixture was prepared by mixing 4.9 ml aliquots of the OV EO emulsion having specified concentration with 100 µl of the microbial inoculum under test. The mixture was vortexed and incubated at room temperature for specified time. It was then vortexed and 0.5 ml volumes were withdrawn and decimally diluted with saline. The numbers of surviving cells were determined by transferring 20 µl portions of each dilution onto the surface of over dried TSA or SDA plates and incubated at 37 °C for 48 h or at 25 °C for 2–5 days for the two media, respectively. The developed colonies were counted and average number of cells was calculated as CFU/ml. Control systems lacking the oil were also included.

#### Effect of OV EO concentration

The same procedure was followed except that the contact time was fixed at 5 min and oil concentration varied between 0 and 10% v/v.

#### Dynamics of the biocidal activity of OV EO

The same procedure was followed except that the number of surviving cells was determined after 5 min–6 h exposure of the test organism to 1% v/v OV EO. All figures were developed using GraphPad Prism^®^ v.5 software.

## Results

### Chemical profile of OV essential oil

**Fig. 1:**
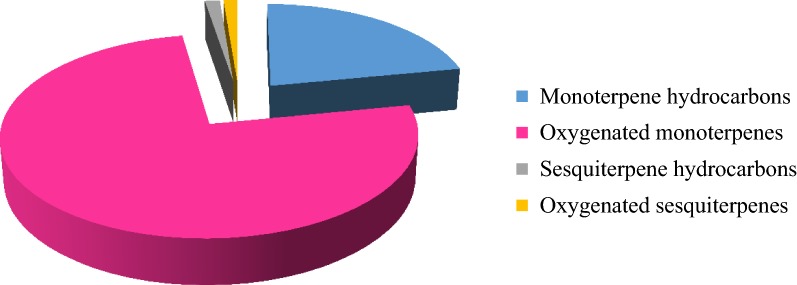
Percentage distribution of classes of constituents identified in OV EO

**Fig. 2:**
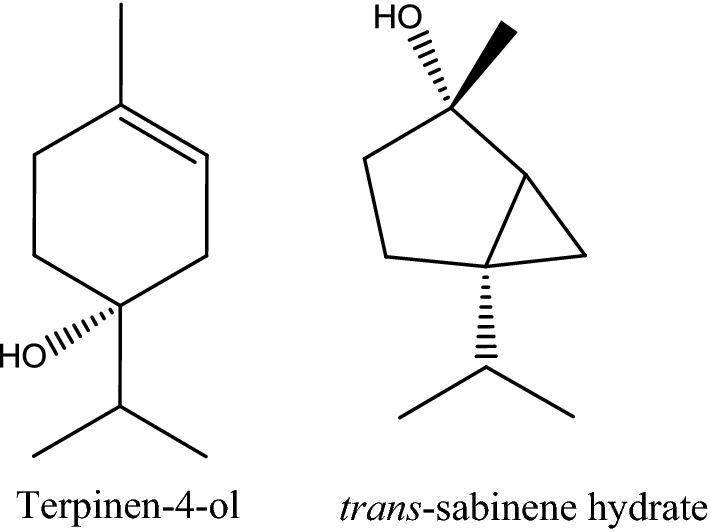
Major identified compounds in OV EO

Hydro-distillation of *O. vulgare* aerial parts yielded 0.85 ± 0.05% v/v pale yellow EO. GC/MS of OV EO allowed the identification of 32 compounds representing 99.94% of the oil (Table 1). Oxygenated monoterpenes predominated other classes of identified components; accounting for 75.41% of the oil (Fig. 1). This is attributed to the presence of the two major identified compounds in this class. These were terpinen-4-ol (38.35%) and *trans*-sabinene hydrate (10.06%) (Fig. 2). Other major identified constituents included α-terpineol (7.32%), α-terpinene (4.51%), *cis*-sabinene hydrate (4.27%) and 4-terpinyl acetate (4.13%).

**Table 1:** Identified constituents in OV EO using GC/MS analysis

No.	Rt	Compound	KI^a^ (calculated)	KI (reported)	Rel. abundance (%)	Methods of identification
		Monoterpene hydrocarbons			21.91	
1	6.065	α-Thujene	918	916	0.90	KI, MS
2	6.240	α-Pinene	931	929	0.22	KI, MS, AT
3	7.330	Sabinene	991	995	2.37	KI, MS
4	7.412	β-Pinene	989	986	0.17	KI, MS, AT
5	7.823	β-Myrcene	1005	1008	0.67	KI, MS
6	8.194	α-Phellandrene	1012	1015	0.22	KI, MS
7	8.541	α-Terpinene	1025	1026	4.51	KI, MS
8	8.804	*p*-Cymene	1036	1039	3.15	KI, MS, AT
9	8.900	β-Phellandrene	1027	1031	1.66	KI, MS
10	9.784	γ-Terpinene	1052	1053	8.04	KI, MS, AT
		Oxygenated monoterpenes			75.41	
11	9.040	1,8-Cineol	1026	1025	0.57	KI, MS, AT
12	10.145	*cis*-Sabinene hydrate	1063	1065	4.27	KI, MS, AT
13	10.663	α-Terpinolene	1079	1082	1.68	KI, MS
14	11.087	trans-Sabinene hydrate	1089	1083	10.06	KI, MS, AT
15	11.757	Camphor	1152	1158	2.99	KI, MS, AT
16	12.323	Linalool	1257	1255	2.06	KI, MS, AT
17	13.409	Terpinen-4-ol	1259	1265	38.35	KI, MS, AT
18	13.839	α-Terpineol	1296	1301	7.32	KI, MS, AT
19	13.955	Estragole	1312	1311	1.06	KI, MS
20	14.340	*cis*-Piperitol	1351	1358	1.03	KI, MS
21	15.567	Linalyl acetate	1389	1398	1.37	KI, MS
22	16.854	4-Terpinyl acetate	1415	1419	4.13	KI, MS
23	16.939	Carvone	1435	1436	0.26	KI, MS, AT
24	17.188	*p*-Cymen-8-ol	1441	1438	0.20	KI, MS
25	19.256	Eugenol	1485	1498	0.06	KI, MS, AT
		Sesquiterpene hydrocarbons			1.36	
26	13.748	α-Humulene	1278	1282	0.17	KI, MS
27	17.793	Bicycloelemene	1578	1572	0.10	KI, MS
28	18.195	γ-Cadinene	1625	1632	0.07	KI, MS
29	20.018	*trans*-Caryophyllene	1412	1410	0.86	KI, MS, AT
30	22.008	Bicyclogermacrene	1494	1489	0.16	KI, MS
		Oxygenated sesquiterpenes			1.26	
31	24.080	Spathulenol	1577	1585	0.57	KI, MS, AT
32	24.182	Caryophyllene oxide	1570	1568	0.69	KI, MS, AT
		Total % of identified compounds			99.94	
		Total number of identified compounds			32.00	

*KI* Kovat’s index, *MS* mass spectral data from wiley libraries, *AT* comparison with authentic compound

^a^KI, Kovat’s index determined on RTX-5MS capillary column

### Antimicrobial studies

#### Effect of emulsifiers on the antimicrobial activity of OV EO

Immiscibility of essential oils with the aqueous culture media creates a methodological problem due to hindrance of proper diffusion. Accordingly, stable o/w emulsions of OV EO were prepared using Tween 20 and Cremphore El. Antimicrobial activity of the prepared oil emulsions was assessed using viable count technique. Results shown in Table 2 reveal that the use of these emulsifiers did not significantly affect the biocidal activity of OV EO Tween 20 was chosen for the experiments due to its relatively lower cost.

**Table 2:** Effect of Tween 20 and Cremophor El on the antimicrobial activity of OV EO

System	Oil conc. (%v/v)	Test microorganism (ATCC no.)		
		*S. aureus* (6538)	*E. coli* (8729)	*C. albicans* (10231)
		Viable count, CFU/ml^a^ (%killing)		
Water^b^	0	5.58 × 10^6^	2.05 × 10^7^	2.58 × 10^6^
	2	5.02 × 10^3^ (99.902)	< 5.00 × 10^1^ (> 99.999)	2.35 × 10^3^ (99.909)
Tween 20, 1%	0	5.26 × 10^6^	9.50 × 10^6^	5.25 × 10^5^
	2	1.18 × 10^5^ (97.8)	<5.00 × 10^1^ (> 99.999)	1.35 × 10^4^ (97.429)
Crempohor El, 1%	0	5.25 × 10^6^	9.55 × 10^6^	5.25 × 10^5^
	2	2.00 × 10^5^ (96.20)	< 5.00 × 10^1^ (> 99.999)	8.30 × 10^3^ (98.40)

^a^Average of three determinations carried out by surface viable method after 5 min at RT (24 °C)

^b^The system was intermittently vortexed for 5 min

#### Biostatic activity of OV EO

Results of agar and broth dilution methods against the tested organisms are shown in Table 3. MIC values of agar dilution method revel that OV EO emulsion had the best activity against *S. aureus*, *Br. bronchiseptica* and all the tested fungi with an MIC 2.36 mg/ml. The least activity was observed against *Ps. aeruginosa* with an MIC > 9.44 mg/ml. Broth dilution method showed better results especially against Gram positive bacteria. The most sensitive organism was *S. aureus* with an MIC 1.18 mg/ml, while the least sensitive one was also *Ps. aeruginosa* with an MIC > 18.88 mg/ml. Agar diffusion method showed that the highest activity was observed against *Br. bronchiseptica*, *Sac. cerevisiae, B. subtilis* and *S. epidermidis* with inhibition zones 38 ± 1.5, 29.5 ± 0.8, 26.9 ± 0.9 and 26.9 ± 1.1 mm, respectively. *Ps. aeruginosa* was the least sensitive organism with inhibition zone 9.3 ± 0.4 mm.

**Table 3:** Biostatic activity of OV EO

Test microorganism (ATCC no.)	Method of assessment			
	Agar dilution	Broth dilution	Agar diffusion	
	MIC (mg/ml)^a^		Inhibition zone, mm^b^	
Ampicillin				
Gram positive organisms				
*B. subtilis* (6633)	2.36	2.36	26.9 ± 0.9	22 ± 0.5
*E. faecalis* (8043)	9.44	2.36	13.6 ± 0.3	18 ± 0.7
*M. lutea* (9341)	4.72	2.36	20.6 ± 0.7	15 ± 0.9
*S. aureus* (6538)	2.36	1.18	20.5 ± 0.6	12 ± 0.4
*S. epidermidis* (12228)	9.44	2.36	26.9 ± 1.1	21 ± 0.8
Gram negative organisms				
*Br. bronchiseptica* (4617)	2.36	2.36	38 ± 1.5	23 ± 0.7
*E. coli* (8729)	4.72	4.72	12.9 ± 0.8	10 ± 0.8
*Ps. aeruginosa* (9027)	> 9.44	> 18.88	9.3 ± 0.4	15 ± 0.4
Fungi				
*C. albicans* (10231)	2.36	2.36	16.1 ± 0.9	15 ± 0.3
*S. cerevisiae* (2601)	2.36	2.36	29.5 ± 0.8	23 ± 0.5
*A. niger* (16404)	2.36	–	–	16 ± 0.8

^a^Minimum Inhibitory concentration, average of three determinations

^b^Average of 10 readings

#### Biocidal activity of OV EO

**Fig. 3:**
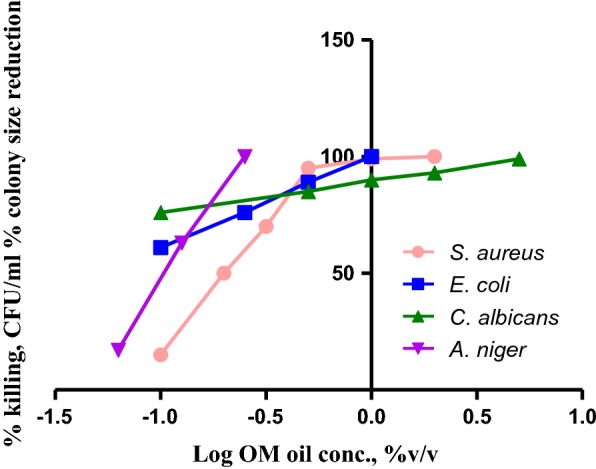
Effect of OV EO on the biocidal activity against four selected organisms

Biocidal activity of OV EO emulsion was studied using viable count technique against four selected organisms; *S. aureus, E. coli. C. albicans and A. niger*. Figure 3 shows the relationship between OV oil concentration and biocidal activity against the four selected organisms. Three of the resultant curves were linear over the entire oil concentration range (0.05–5.0% v/v) tested. The curve of *S. aureus* was bilinear having a much steeper part at oil concentration ≤ 1% v/v. The curves of the four tested organisms displayed different slopes; that of *A. niger* was the most responsive while that of *C. albicans* was the least. OV oil emulsion concentrations causing 50% and 75% microbial growth inhibition were calculated from the dose response curve obtained in Fig. 3 (Table 4).

**Table 4:** Concentrations of OV EO causing 50% and 75% microbial growth inhibition

Test microorganism	Oil conc. causing 50% microbial growth inhibition (%v/v)	Oil conc. causing 75% microbial growth inhibition (%v/v)
*E. coli*	0.08	0.21
*S. aureus*	0.17	0.34
*A. niger*	0.11	0.15
*C. albicans*	0.07	0.09

Concentrations are calculated from dose–response curve (Fig. 1)

#### Dynamics of the biocidal activity of OV EO

**Fig. 4:**
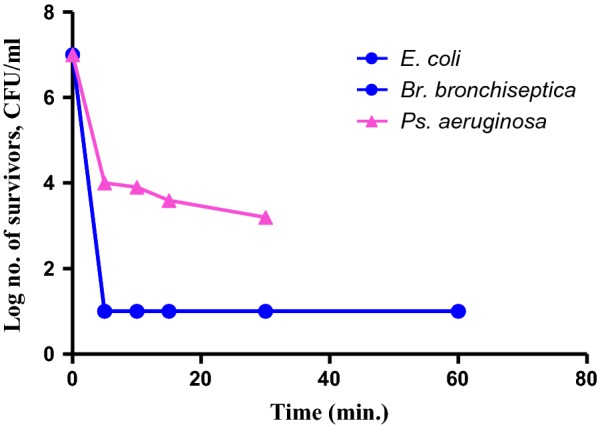
Biocidal dynamics of OV EO (1% v/v) against three Gram negative bacteria

**Fig. 5:**
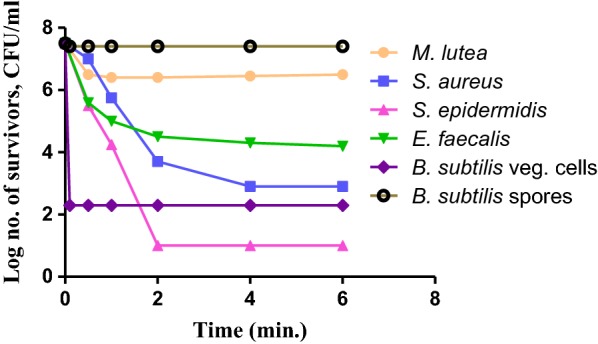
Biocidal dynamics of OV EO (1% v/v) against three Gram positive bacteria

**Fig. 6:**
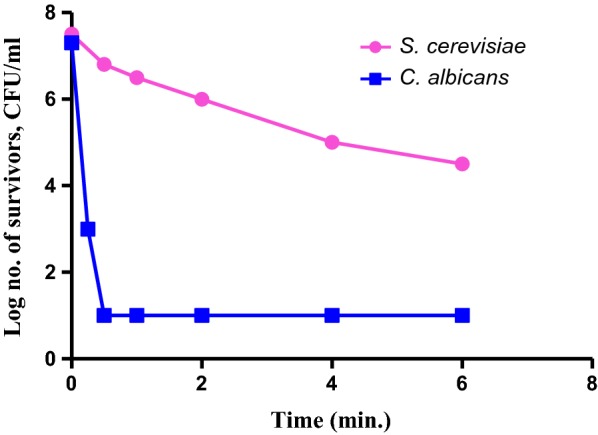
Biocidal dynamics of OV EO (1% v/v) against two yeast strains

The dynamics of OV EO emulsion biocidal activity against 10 microorganisms are presented in Figs. 4, 5, and 6. These figures relate the level of biocidal activity of 1% v/v of OV EO emulsion vs. time of exposure of the test organisms.

Figure 4 shows the dynamics of the bactericidal activity of the oil against 3 Gram negative bacteria. The response of both *E. coli* and *Br. bronchiseptica* was quite rapid; more than 99.999% of the exposed cells (ca 10^7^ CFU/ml) were killed within 5 min contact with the oil (1% v/v) and no re-growth was detected after that. In contrast, the response of *Ps. aeruginosa* was distinctly different, exhibiting bilinear response vs. time. The first part of dynamic response was relatively rapid leading to 3 log reductions in the number of cells surviving the first 5 min of incubation. Thereafter, killing rate decreased markedly and, at the end of the experiment (6 h), ca 10^4^ CFU/ml was still living.

Figure 5 shows the dynamics of biocidal of 1% v/v oil emulsion against 5 Gram positive bacteria. Among these*, B. subtilis* spores were completely insensitive followed by *M. lutea* which displayed only small (1 log) initial drop in the number of cells surviving after 30 min contact with the oil with no further drop*. E. faecalis* showed intermediate response for ca 2 logs. Reduction in viable count took place within 2 h contact with the oil and further insignificant reduction occurred during the remaining 4 h of incubation. A sigmoid curve was observed with *S. aureus*. It showed ca 4 logs reduction in the number of survivor by the end of the first 2 h of contact with the oil followed by ca 1.5 log further reductions by the last hour of inoculation (6 h). The dynamic of biocidal response of *S. epidermidis* was quite rapid showing more than 99.999% killing by the end of the first 2 h of contact with the oil and no further growth was noticed. When *B. subtilis* culture was exposed to 1% v/v of OV EO, ca. 5.5 log reduction in the inoculated cells (7.5 logs) took place quite rapidly (15 min) and remained the same for the rest of the experiment (6 h). These survivors were found to be entirely spores; confirming the non-sporicidal activity of the OV EO.

The dynamics of the biocidal activity of 1% v/v OV EO emulsion against *C. albicans* and *S. cerevisiae* (yeast strains) are shown in Fig. 6. The latter strain was sensitive to the oil since practically all the exposed cells (ca. 10^7^ CFU/ml) were killed during the first 30 min of contact. On the other hand*, C. albicans* cells exhibited much slower dynamics and, at the end of the experiment (6 h), out of 7.5 logs of exposed cells, ca. 4.5 logs were still surviving.

## Discussion

The essential oil composition of *Origanum* genus has been extensively studied, and has shown a wide variation between its different species. The variation in composition may even be present within the same species collected during different seasons or from different geographical areas. In the present study, GC/MS analysis of the hydro-distilled OV EO allowed the identification of 32 compounds representing 99.94% of the oil. The major identified compound was the oxygenated monoterpene; terpinen-4-ol. Terpinen-4-ol was also the major identified constituent of the OV EO grown in India, Israel and Venezuela (Govindarajan et al. 2016; Ramos et al. 2011; Ravid et al. 1987; Vera and Chane-Ming 1999). It is also a major constituent in the oil of other *Origanum* species like *O. ramonese*, *O. scabrum* and *O. microphyllum* (Aligiannis et al. 2001; Danin et al. 1997). Results of the biostatic studies on OV EO using agar and broth dilution methods showed that *S. aureus* was the most sensitive organism to OV EO emulsion., with an MIC 0.125% v/v. Other studies also report a high sensitivity of *S. aureus* to OV EO with an MIC ranging 0.31–10 µg/ml (Honório et al. 2015; Pesavento et al. 2015; Tavares et al. 2015). Agar diffusion method showed that the highest activity was observed against *Br. bronchiseptica*, *Sac. cerevisiae, B. subtilis* and *S. epidermidis* with inhibition zones 38 ± 1.5, 29.5 ± 0.8, 26.9 ± 0.9 and 26.9 ± 1.1 mm, respectively. It should be also noted that the Gram negative *Pseudomonas aeruginosa* was the least sensitive organism, as shown by the results obtained by all the used tests, while *A. niger* was completely insensitive as shown by the broth dilution and agar diffusion methods. On the other side, study of the biocidal activity of OV EO emulsion using viable count technique showed that 75% of *C. albicans* colonies died at a concentration of 0.09% v/v of the oil emulsion in only 5 min. This is supported by several studies that show a high activity of OV EO against different *Candida* species, especially vaginal and oral *Candida* (Bhat et al. 2018; Cleff et al. 2010). Studying the dynamics of 1% v/v OV EO emulsion over a period of 6 h revealed that *E. coli, B. subtilis, S. epidermidis* and *S. cerevisiae* had the fastest response, while all other organisms had more or less intermediate response except for *B. subtilis* spores which were completely insensitive to the oil emulsion. This markedly strong antimicrobial activity may be attributed to the presence of the alcohol terpinene-4-ol in the EO. This compound has proven a strong antimicrobial activity against several organisms like *B. subtilis, Bacteroides fragilis, Candida* spp., *Clostridium perfringens, E. faecalis, E. coli, Lactobacillus acidophilus, Moraxella catarrhalis, Mycobacterium smegmatis, Ps. aeruginosa, Serratia marcescens* and *S. aureus* (Carson and Riley 1995; Hammer et al. 2012; Mondello et al. 2006). Terpinen-4-ol is also the major compound present in tea tree oil which is known for its broad spectrum antimicrobial properties (Carson et al. 2006; Lee et al. 2013; Sharifi-Rad et al. 2017). The suggested mechanism of action of terpinen-4-ol may be due to compromising of the cytoplasmic membrane (Carson et al. 2002), or penetration of organelle membrane inducing deformation, damage and eventually microbial cell death (Li et al. 2016).

In conclusion, results obtained in this study showed that OV EO possesses strong antibacterial and antifungal activities, especially against *S. aureus* and *Br. bronchiseptica* which may be attributed mainly to the presence of terpinen-4-ol in its essential oil composition.

## Abbreviations

*B. subtilis*: *Bacillus subtilis*
*E. faecalis*: *Enterococcus faecalis*
*M. lutea*: *Micrococcus lutea*
*S. aureus*: *Staphylococcus aureus*
*S. epidermidis*: *Staphylococcus epidermidis*
*Br. bronchiseptica*: *Brodetella bronchiseptica*
*E. coli*: *Escherichia coli*
*Ps. aeruginosa*: *Pseudomonas aeruginosa*
*C. albicans*: *Candida albicans*
*S. cerevisiae*: *Saccharomyces cerevisiae*
*A. niger*: *Aspergillus niger*
*OV*: *Origanum vulgare*
*O. vulgare*: *Origanum vulgare*
GC/MS: gas chromatography/mass spectrometry
EO: essential oil
MAPs: medicinal and aromatic plants
ATCC: American Type Culture Collection
MIC: minimum inhibitory concentration
TSA: trypticase soy agar
SDA: Sabouraud Dextrose Agar
id: internal diameter
v/v: volume/volume
h: hour
min.: minutes
